# Benign Biliary Tumors and Precursor Neoplasms: An Updated Clinicopathological and Molecular Review Based on the 2026 WHO Classification

**DOI:** 10.3390/biomedicines14071548

**Published:** 2026-07-10

**Authors:** Joon Hyuk Choi

**Affiliations:** Department of Pathology, Yeungnam University College of Medicine, 170 Hyeonchung-ro, Namgu, Daegu 42415, Republic of Korea; joonhyukchoi@ynu.ac.kr

**Keywords:** bile duct adenoma, mucinous cystic neoplasm, intraductal papillary neoplasm of the bile ducts, pathology, diagnosis, molecular genetics, classification

## Abstract

Benign biliary tumors and precursor neoplasms of the biliary tract are a heterogeneous group of uncommon neoplasms with significant clinical and diagnostic implications. Their importance lies in their variable malignant potential and morphological and molecular similarities to pancreatic neoplasms. The sixth edition of the World Health Organization Classification of Digestive System Tumours (WHO DST6), published online in 2026, introduced major revisions to the classification of these entities. According to WHO DST6, benign tumors and precursor neoplasms of the liver and intrahepatic bile ducts include bile duct adenoma, biliary adenofibroma, and mucinous cystic neoplasm, whereas those of the gallbladder and extrahepatic bile ducts include biliary intraepithelial neoplasia, intraductal papillary neoplasm of the bile ducts, intraductal tubulopapillary neoplasm of the bile ducts, intraductal oncocytic papillary neoplasm of the bile ducts, and mass-forming intracholecystic neoplasm. Despite these advances, their histological and molecular heterogeneity continues to pose significant diagnostic challenges, particularly in distinguishing them from malignant biliary tumors on limited biopsy specimens and in recognizing early invasive lesions. This review summarizes the clinicopathological and molecular features of benign biliary tumors and precursor neoplasms, emphasizing differential diagnosis and key updates introduced in WHO DST6.

## 1. Introduction

The sixth edition of the World Health Organization (WHO) Classification of Digestive System Tumours (WHO DST6), published online in 2026, introduced substantial revisions to the classification of benign biliary tumors and precursor neoplasms [1,2]. According to WHO DST6, benign biliary tumors and precursor neoplasms of the liver and intrahepatic bile ducts include bile duct adenoma (BDA), biliary adenofibroma (BAF), and mucinous cystic neoplasm (MCN), whereas those of the gallbladder and extrahepatic bile ducts include biliary intraepithelial neoplasia (BilIN), intraductal papillary neoplasm of the bile ducts (IPNB), intraductal tubulopapillary neoplasm (ITPN) of the bile ducts, intraductal oncocytic papillary neoplasm (IOPN) of the bile ducts, and mass-forming intracholecystic neoplasm (ICN). The evolution of the WHO classification for these entities is summarized in Table 1.

Despite the rarity of benign biliary tumors and precursor neoplasms, marked geographic variation in incidence, variable malignant potential, and pathogenetic overlap with pancreatic precursor neoplasms underscore their clinical significance [3,4,5]. Noninvasive epithelial neoplasms of the biliary tract and pancreas share common differentiation pathways and recurrent genetic alterations, supporting a unified model of stepwise pancreaticobiliary tumorigenesis [6,7,8].

Despite advances in molecular characterization and classification systems, these lesions remain diagnostically challenging. Substantial histological overlap among reactive, benign, preinvasive, and early invasive lesions—compounded by molecular heterogeneity—further complicates diagnostic interpretation. Although WHO DST6 offers an integrated clinicopathological and molecular framework, its application in routine practice necessitates a systematic approach.

This review summarizes benign biliary tumors and precursor neoplasms according to WHO DST6, emphasizing diagnostic criteria, molecular pathogenesis, and differential diagnosis. Lesions of the liver and intrahepatic bile ducts are addressed first, followed by those of the gallbladder and extrahepatic bile ducts, to provide a practical, diagnostically oriented framework for pathologists and clinicians.

A literature search of PubMed and Google Scholar was conducted for articles published through April 2026 using keywords including “biliary precursor neoplasms,” “IPNB,” and other entities recognized in the WHO DST6. Literature selection prioritized high-quality original studies, expert consensus statements, and molecular landscape investigations to provide a comprehensive overview of the current evidence.

## 2. Benign Biliary Tumors and Precursor Neoplasms in the Liver and Intrahepatic Bile Ducts

### 2.1. Bile Duct Adenoma

#### 2.1.1. Clinical Features

BDA is a benign nodular lesion characterized by the proliferation of small bile ducts. It is detected in approximately 0.6% of autopsy cases [9] and 2.4% of liver resection specimens [10]. BDA typically presents as a solitary subcapsular nodule (approximately 90% of cases), although it may occur anywhere in the liver. It predominantly affects adults and shows no sex predilection. On imaging, BDA typically presents as a small, well-circumscribed, hypovascular nodule in the subcapsular region of the liver. The pathogenesis of BDA remains uncertain. The proposed mechanisms include reactive changes secondary to focal injury [11] and an origin from peribiliary glands [12]. Atypical features, including larger size and microcystic changes, have been reported and may raise concern for malignant transformation [13,14].

#### 2.1.2. Molecular Features

*BRAF* p.V600E mutations have been identified in approximately 53% of BDAs, supporting a neoplastic rather than purely reactive nature in a subset of cases [15,16].

#### 2.1.3. Pathological Features

**Figure 1:**
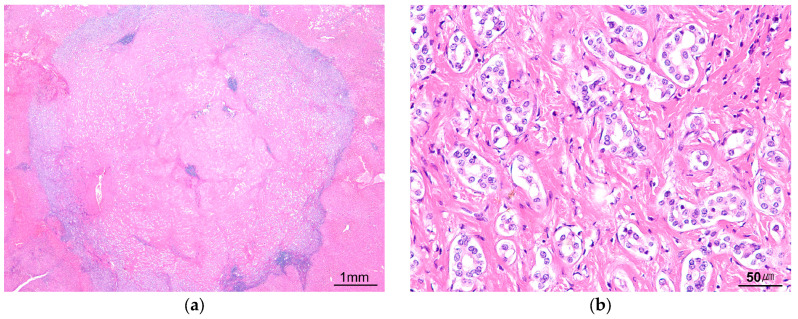
Bile duct adenoma. (**a**) A well-circumscribed, unencapsulated lesion is present (hematoxylin and eosin stain, ×1). (**b**) Uniform tubules lined by a single layer of cuboidal epithelial cells are embedded in hyalinized fibrous stroma, without cytological atypia or mitoses (hematoxylin and eosin stain, ×200). All images are representative gross and histopathological findings selected from the author’s institutional archives for illustrative purposes only. No new data analysis or previously unpublished data are included.

Grossly, BDA typically presents as a small (<10 mm), white, firm, well-circumscribed, unencapsulated nodule that is usually solitary, although multiple nodules may occur. Histologically, it is characterized by a disordered proliferation of uniform small tubules embedded within a fibrous stroma, without infiltrative borders (Figure 1). The tubules are lined by bland cuboidal epithelial cells resembling bile ductules. Entrapped portal tracts are frequently present. The stroma commonly shows collagenization and chronic inflammatory infiltrates. Immunohistochemically, ductal cells express cytokeratin 7 (CK7) and CK19. Positivity for MUC5AC and MUC6 is observed in approximately 80–90% of cases [17]. Morphological variants include mucin production, collagenized stroma, and clear cell or oncocytic features [18,19]. The oncocytic variant is characterized by abundant eosinophilic cytoplasm and centrally located round nuclei.

#### 2.1.4. Differential Diagnosis

Differential diagnoses include ductular reaction (DR), von Meyenburg complex (VMC; biliary microhamartoma), and adenocarcinoma, including primary intrahepatic cholangiocarcinoma (iCCA) and metastatic tumors. DR is typically ill-defined and non-nodular. However, in cirrhosis, it may replace hepatocytes in areas of parenchymal extinction, resulting in a vaguely nodular lesion. VMCs are typically smaller than BDAs (usually <5 mm) and are characterized by irregular, angulated ducts within abundant fibrous stroma, often containing intraluminal bile. In contrast, BDA typically lacks cystic change and intraluminal bile. Small-duct type intrahepatic cholangiocarcinoma (SD-iCCA), particularly the cholangiolocellular subtype, may closely mimic BDA by demonstrating well-differentiated small ductular structures with only mild cytologic atypia. However, SD-iCCA can be distinguished from BDA by its larger size, more complex architecture, and invasive growth. Immunohistochemistry (IHC) for Ki-67, p53, EZH2, and p16 may be diagnostically useful, particularly in challenging cases [20,21]. BDAs demonstrate a low Ki-67 index (typically <10%) and lack strong p53 expression. Although EZH2 is expressed in carcinomas, it is typically absent in BDAs. p16 expression is typically retained in BDAs, whereas loss of expression is frequently observed in carcinomas. BDAs may also demonstrate C-reactive protein expression and albumin in situ hybridization (ISH) positivity [22].

#### 2.1.5. Key Updates in the 6th WHO Classification

In WHO DST6, BDA has undergone conceptual refinement: the term “peribiliary gland hamartoma” is no longer recommended, and accumulating evidence supports a neoplastic nature in a subset of cases, particularly those harboring *BRAF* p.V600E mutations. Furthermore, the recognized anatomical spectrum has been expanded to encompass lesions beyond the hepatic subcapsular region.

### 2.2. Biliary Adenofibroma

#### 2.2.1. Clinical Features

BAF is a rare benign intrahepatic epithelial neoplasm with a solid and microcystic architecture, composed of variably dilated tubules lined by nonmucinous biliary epithelium within a fibrotic stroma. BAF is extremely rare, with published data limited to case reports and small series [23,24,25,26]. Affected patients range in age from 23 to 83 years (median, 57 years), with a slight female predominance. BAF has no predilection for a specific hepatic location. Patients may present with abdominal pain; however, incidental detection is common. On imaging, BAF appears as a well-circumscribed mass with a sponge-like appearance, reflecting mixed solid and microcystic components. Malignant transformation has been reported in approximately 50% of cases [24,27].

#### 2.2.2. Molecular Features

Recurrent hotspot mutations are absent in BAF; however, the presence of multiple clonal cytogenetic alterations supports a neoplastic nature. *CCND1* and *ERBB2* amplifications have been identified and may be associated with biological aggressiveness. *CDKN2A* (p16) and/or *NRAS* mutations have been reported in rare cases, particularly those associated with malignant transformation [28,29].

#### 2.2.3. Pathological Features

Grossly, BAF presents as a solitary, well-circumscribed but unencapsulated, round to oval mass (17–160 mm). On cut section, the tumor shows mixed solid and microcystic (sponge-like) areas, with occasional macrocysts (>10 mm). Histologically, it is composed of acini, tubules, and cysts embedded within an abundant fibrotic stroma. The tubules are often dilated and branched, and the cysts vary in size, occasionally showing intraluminal polypoid projections. The lining epithelium is single-layered and nonmucinous, consisting of cuboidal to low columnar cells, with amphophilic cytoplasm and bland nuclei; focal epithelial flattening and apocrine snouts may be present. The stroma is collagenous and relatively hypocellular, containing bland spindle-shaped myofibroblasts with patchy mononuclear inflammatory infiltrates. The epithelium demonstrates a biliary immunophenotype (positivity for EMA, CK7, CK19, and CA19-9), and the Ki-67 proliferation index is low (<10% in the epithelium; <1% in the stroma). Premalignant dysplasia is observed in approximately 50% of cases and is characterized by nuclear atypia (elongation, hyperchromasia, and pseudostratification) [24] and increased architectural complexity (intracystic, papillary, and cribriform growth patterns) [30,31]. Carcinoma arising from BAF most commonly resembles conventional adenocarcinoma, consistent with SD-iCCA [32,33]; rare cases of microcystic-cribriform carcinoma have also been reported [34].

#### 2.2.4. Differential Diagnosis

Differential diagnoses include VMC and the tubulocystic variant of SD-iCCA. Small BAFs may overlap with large VMCs. Smaller lesions (<1 cm) are typically consistent with VMC, whereas lesions > 2 cm favor BAF; the presence of prominent micro- or macrocysts further supports a diagnosis of BAF. Tubulocystic SD-iCCA may exhibit BAF-like areas [35]. Shared chromatin-remodeling gene alterations (e.g., *ARID1A*, *BAP1*, *IDH2,* and *PBRM1*) or an *FGFR2*::*MCU* fusion gene in both adenocarcinoma and BAF-like components support the neoplastic and potentially malignant nature of the BAF-like component [35,36]. The presence of a conventional SD-iCCA component or cytologic atypia favors tubulocystic iCCA, whereas BAF is typically well-circumscribed, with hypocellular fibrotic stroma and bland cytology.

#### 2.2.5. Key Updates in the 6th WHO Classification

In WHO DST6, BAF is more precisely defined as a benign intrahepatic epithelial neoplasm with a malignant transformation rate of approximately 50%. Accumulating molecular data, including *NRAS* mutations alongside *CDKN2A* alterations, further expands the spectrum of genetic changes associated with malignant transformation. Furthermore, the morphological overlap with tubulocystic SD-iCCA highlights the importance of thorough histological assessment to exclude invasive carcinoma.

### 2.3. Mucinous Cystic Neoplasm

#### 2.3.1. Clinical Features

Mucinous cystic neoplasm (MCN) is a rare, cyst-forming epithelial neoplasm characterized by mucin-producing cuboidal to columnar epithelium overlying ovarian-type stroma, with no communication with the bile ducts. Its incidence is approximately 1 per 20,000–100,000 person-years [37,38], and it accounts for approximately 10% of all hepatic cysts [39]. MCN occurs almost exclusively in women, and the presence of ovarian-type stroma supports a hormonal influence [37]. The mean patient age is 52 years [39], with patients harboring invasive carcinoma presenting at an older age (mean, 61 years) than those with noninvasive MCN (mean, 50.4 years) [40]. Tumors are predominantly intrahepatic [37,40,41] and typically solitary, most commonly arising in the left lobe (particularly segment IV). Rare cases arising in the gallbladder have been reported [42,43]. Clinically, patients usually present with abdominal pain or distension. Elevated CA19-9 and CEA levels in serum or cyst fluid may help distinguish MCN from non-neoplastic cysts [44,45]. On imaging, MCN characteristically appears as a multilocular cystic mass with internal septations; wall thickening, irregular septa, or papillary projections should raise suspicion for associated invasive carcinoma [46,47]. Prognosis following complete surgical resection is excellent [38,48]. Associated invasive carcinoma is uncommon (approximately 6%), and although it confers a worse prognosis than noninvasive MCN, outcomes remain more favorable than those of conventional iCCA [40,48].

#### 2.3.2. Molecular Features

*KRAS* mutations are identified in approximately 20% of MCNs, whereas *GNAS, RNF43*, and *PIK3CA* are typically wild-type [40,41,49]. *KRAS* mutations are uncommon in low-grade dysplasia (approximately 5%) but more frequent in high-grade dysplasia, supporting their role in neoplastic progression. *KRAS*-mutated MCNs more often exhibit multilocular cystic morphology and increased expression of MUC1, MUC2, and MUC5AC compared with *KRAS*-wild-type tumors [41]. Progressive telomere shortening has been observed during progression to invasive carcinoma [50]. Primordial germ cells may represent a shared cell of origin for pancreatic MCN and mucinous ovarian tumors [51], a concept potentially applicable to hepatobiliary MCN given their morphological and molecular similarities.

#### 2.3.3. Pathological Features

**Figure 2:**
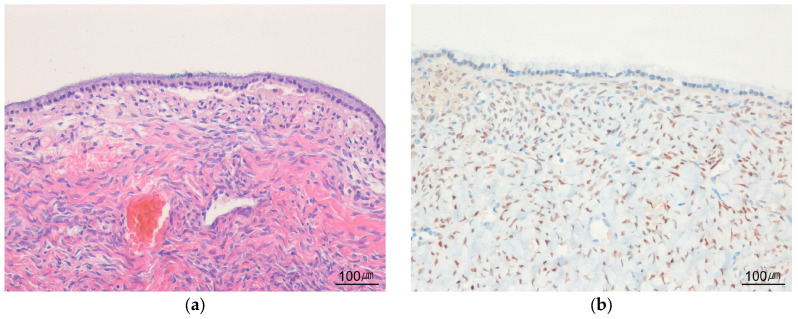
Mucinous cystic neoplasm with low-grade intraepithelial neoplasia. (**a**) The cyst is lined by columnar epithelial cells with mucinous cytoplasm, with underlying ovarian-type spindle cell stroma (hematoxylin and eosin stain, ×100). (**b**) The ovarian-type stromal cells are positive for progesterone receptor (PR), while the overlying epithelium is negative for PR (immunohistochemical stain, ×100).

**Figure 3:**
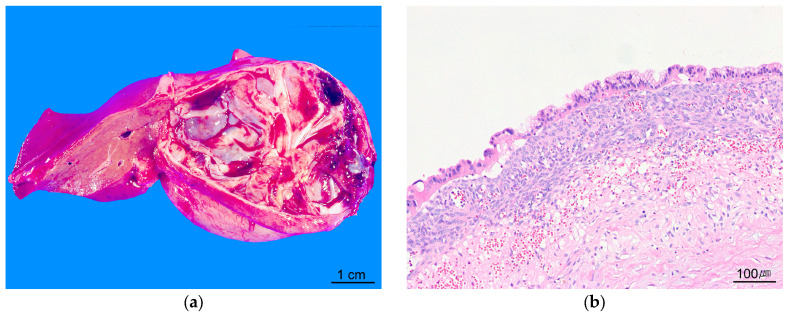

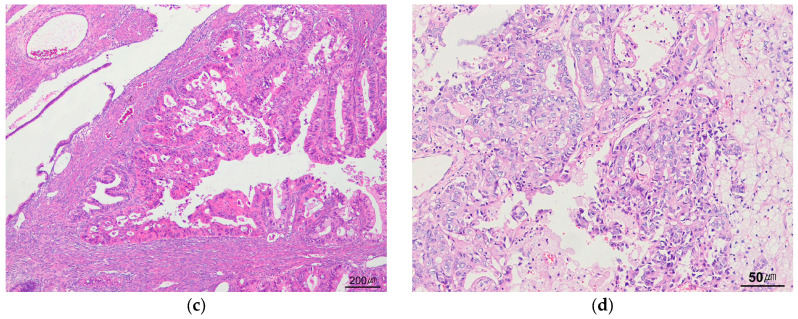
Mucinous cystic neoplasm with associated invasive adenocarcinoma. (**a**) A multiloculated cystic tumor is present in the left hepatic lobe, with internal septations containing mucinous and hemorrhagic material. (**b**) The tumor is lined by low-grade mucinous epithelium with underlying ovarian-type stroma (hematoxylin and eosin stain, ×100). (**c**) A transition from low-grade to high-grade dysplasia with marked cytological atypia is present (hematoxylin and eosin stain, ×100). (**d**) An associated invasive adenocarcinoma component is identified within the mucinous cystic neoplasm (hematoxylin and eosin stain, ×100).

Grossly, MCNs are well-circumscribed, multilocular cystic lesions (50–290 mm; mean, 110 mm) [38,40,41,52], typically separate from the biliary tree. The inner surface is smooth or trabeculated, occasionally with papillary projections, and the cysts contain mucinous, clear, or hemorrhagic fluid. Associated invasive carcinoma may appear as a solid, gray-white mural nodule or mass. Histologically, the cysts are lined by columnar, cuboidal, or flattened epithelial cells with mucinous or pale eosinophilic cytoplasm and basally oriented nuclei, occasionally forming papillary structures (Figure 2a). Approximately 50% show nonmucinous biliary-type epithelium, and scattered neuroendocrine cells are identified in approximately 50% of cases [40,53]. The ovarian-type stroma is hypercellular and often rimmed by fibrous tissue [40,41], with focal luteinization or hyalinization in some cases. Degenerative changes, including hemorrhage, calcification, and necrosis, and associated inflammation, are common [40]. Immunohistochemically, the epithelial cells are positive for CK7, CK8, CK18, CK19, EMA, CEA, and MUC5AC [40] and may show gastric or intestinal differentiation or squamous metaplasia. Stromal cells are positive for ER, PR, and α-inhibin (Figure 2b) [40,42]. Most MCNs exhibit low-grade dysplasia; high-grade dysplasia, characterized by marked architectural atypia and increased mitotic activity, is uncommon [40]. Invasive carcinoma occurs in approximately 6% of cases, typically as tubular or tubulopapillary adenocarcinoma with desmoplastic stroma, resembling large-duct type intrahepatic cholangiocarcinoma (LD-iCCA) (Figure 3) [40,41].

#### 2.3.4. Differential Diagnosis

Differential diagnoses include solitary bile duct cyst (simple cyst), ciliated foregut cyst, endometriotic cyst, and IPNB. Solitary bile duct cysts are typically unilocular and lack ovarian-type stroma. Ciliated foregut cysts are distinguished by their ciliated epithelium and lack ovarian-type stroma. Endometriotic cysts contain endometrial glands and stroma, and ER and PR are positive in both epithelium and stromal cells of endometriotic cysts, whereas in MCNs, ER and PR expression is restricted to stromal cells [54]. Unlike MCNs, IPNBs communicate with the bile ducts and lack ovarian-type stroma. The presence of ovarian-type stroma is a defining diagnostic criterion for MCN; its absence precludes the diagnosis.

#### 2.3.5. Key Updates in the 6th WHO Classification

In WHO DST6, the definition of MCN has been refined as a noninvasive cyst-forming epithelial neoplasm with ovarian-type stroma and no communication with the bile ducts. The grading system has been simplified to a two-tiered scheme (low- and high-grade dysplasia), enhancing diagnostic reproducibility. Molecularly, *KRAS* mutations are recognized as progression-associated events that occur more frequently in high-grade dysplasia. The absence of *GNAS* mutations and the presence of progressive telomere shortening support a stepwise precursor-to-carcinoma progression model.

Table 2 summarizes the clinicopathological and molecular features of benign biliary tumors and precursor neoplasms in the liver and intrahepatic bile ducts.

## 3. Benign Biliary Tumors and Precursor Neoplasms in the Gallbladder and Extrahepatic Bile Ducts

### 3.1. Biliary Intraepithelial Neoplasia

#### 3.1.1. Clinical Features

BilIN is a microscopic, noninvasive flat or (micro)papillary epithelial neoplasm involving the large bile ducts and gallbladder. It may involve the entire extrahepatic biliary tree and the large intrahepatic bile ducts, while generally sparing the small terminal intrahepatic bile ducts. BilIN is usually detected incidentally in resected specimens. BilIN is radiologically occult as a microscopic lesion, although subtle mucosal thickening or irregularity may be detected on high-resolution cholangiography. It is identified in the surrounding mucosa of up to 80% of invasive gallbladder carcinomas [55,56]. In endemic regions, low-grade BilIN is observed in up to 15% of gallbladders with cholelithiasis, whereas high-grade BilIN occurs in 1.5–3.5% of cases [57]. The prevalence of high-grade BilIN parallels that of invasive biliary carcinoma [57] and shows marked geographic variation, occurring in up to 5% of cases in East Asia compared with <0.1% in North America [57,58]. BilIN is associated with hepatolithiasis, cholelithiasis, primary sclerosing cholangitis (PSC) [59,60], choledochal cyst [61], anomalous union of the pancreaticobiliary ducts (AUPBD) [62], and parasitic infections (e.g., *Opisthorchis viverrini* and *Clonorchis sinensis*) [63].

#### 3.1.2. Molecular Features

Chronic inflammation plays a central role in the pathogenesis of BilIN [64]. In the gallbladder, persistent inflammation induces oxidative stress, which causes DNA damage and genomic instability. The effects of this are exacerbated by defects in DNA repair genes (e.g., *ATM* and *BRCA2*) and by epigenetic alterations, including the hypermethylation of tumor suppressor gene promoters such as *FHIT*, *APC*, *CDKN2A*, *CDH1*, *RB1*, *TP53*, and *DCC*. Genomic studies have identified recurrent mutations in several genes (e.g., *CTNNB1, ARID2*, *TP53*, *ERBB3*, *NFE2L2*, *PIK3CA*, *SMARCA4*, *KRAS*, and *SMAD4*) [65,66], with *CTNNB1* mutations implicated in the progression to invasive carcinoma [65]. In the large bile ducts, *KRAS* mutations are often early events, present in 30–40% of low-grade BilIN, whereas *TP53* alterations occur later in tumor progression [67,68,69].

#### 3.1.3. Pathological Features

**Figure 4:**
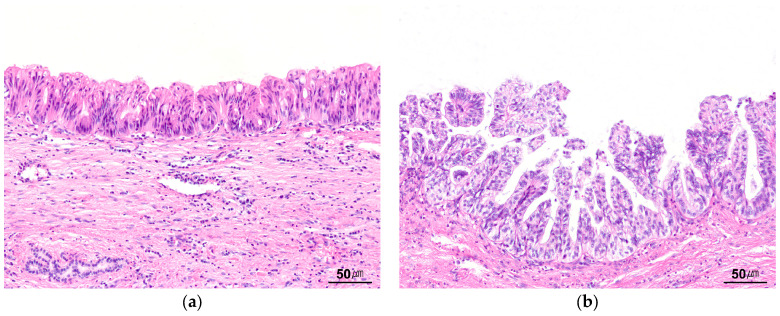
Biliary intraepithelial neoplasia (BilIN). (**a**) Low-grade BilIN shows nuclear pseudostratification with an increased nuclear-to-cytoplasmic (N:C) ratio and nuclear hyperchromasia (hematoxylin and eosin stain, ×200). (**b**) High-grade BilIN shows irregular nuclei, a high N:C ratio, and loss of polarity. Epithelial budding is present (hematoxylin and eosin stain, ×200).

Grossly, BilIN lesions are typically inapparent, although subtle mucosal thickening or textural changes may be present. Histologically, BilIN is graded as low- or high-grade based on cytoarchitectural atypia [70]. This two-tiered system replaces the previous BilIN-1/2 (low-grade) and BilIN-3 (high-grade) classification [70,71]. Low-grade BilIN shows mild cytological atypia, predominantly flat growth, nuclear pseudostratification, increased nuclear-to-cytoplasmic ratio, hyperchromasia, and preserved basal polarity (Figure 4a). High-grade BilIN exhibits complex architecture (micropapillary or tall papillary), loss of polarity, marked nuclear atypia, and frequent mitoses (Figure 4b) [72]. Low-grade BilIN is usually focal and rarely associated with invasive carcinoma, whereas high-grade BilIN is often extensive and frequently associated with invasive carcinoma [55,73,74]. Phenotypes (biliary, intestinal, gastric) lack clear clinical significance and do not necessarily correlate with invasive components [75]. The epithelium adjacent to BilIN lesions is often inflamed and reactive, frequently showing intestinal or foveolar-type gastric metaplasia [66,76,77,78].

#### 3.1.4. Differential Diagnosis

Differential diagnoses include reactive epithelial atypia (REA) and invasive adenocarcinoma. The distinction between BilIN and REA can be challenging. REA typically involves both the surface epithelium and intramural glands, characterized by attenuated basophilic cells, intercellular clefts, occasional nuclear molding, fine chromatin, small to conspicuous nucleoli, and brisk mitotic activity. A gradual transition from normal to atypical epithelium favors REA rather than BilIN. Immunohistochemically, p53 overexpression supports the diagnosis of BilIN [79,80,81]. MUC5AC and S100P expression may increase with higher-grade dysplasia [82,83,84,85]; however, their variable expression limits diagnostic utility. High-grade BilIN in the gallbladder may arise from or extend into Rokitansky–Aschoff sinuses, mimicking invasive adenocarcinoma [86,87]. In this setting, the absence of a desmoplastic stromal reaction and irregular stromal invasion, along with confinement of the lesion to preexisting sinus structures, favors BilIN over invasive adenocarcinoma.

#### 3.1.5. Key Updates in the 6th WHO Classification

In WHO DST6, the etiological spectrum has been expanded to include parasitic infections. The updated classification emphasizes chronic inflammation and incorporates DNA repair defects, epigenetic alterations, and recurrent somatic mutations into the molecular framework of BilIN. Notably, *CTNNB1* mutations have been implicated as potential drivers of progression to invasive carcinoma.

### 3.2. Intraductal Papillary Neoplasm of the Bile Ducts

#### 3.2.1. Clinical Features

IPNB is a grossly visible, noninvasive epithelial neoplasm of the large intrahepatic and extrahepatic bile ducts, characterized by intraluminal papillary or tubular proliferations of mucin-producing columnar cells. Its prevalence shows marked geographic variation, accounting for 10–38% of bile duct tumors in East Asia but only 7–11% in Western countries [88,89,90,91]. Patients are typically aged 42–80 years, with a slight male predominance [92,93]. The anatomical distribution varies between intrahepatic and extrahepatic sites [93,94]. IPNB appears as an intraluminal polypoid or papillary mass associated with bile duct dilatation on cross-sectional imaging. On cholangiography, IPNB typically presents as intraluminal filling defects, although lesions may occasionally be occult [95,96]. Clinically, patients typically present with recurrent abdominal pain, jaundice, and cholangitis [4,93,96,97]. The etiology is not fully defined; however, risk factors include PSC [98], hepatolithiasis, and liver fluke infection (*Clonorchis sinensis* and *Opisthorchis viverrini*)—particularly in East Asian populations [99,100,101]—and pancreaticobiliary maljunction (PBM) [102,103]. IPNB progresses from low-grade to high-grade dysplasia and may evolve into invasive carcinoma [6,93,100]. IPNB-associated invasive carcinoma is associated with better survival than conventional CCA.

#### 3.2.2. Molecular Features

IPNB develops through the stepwise accumulation of somatic alterations, including *KRAS* mutations, p16 loss, and inactivating *TP53* mutations [6,104,105]. Molecular heterogeneity correlates with histological subtypes. *GNAS* mutations are identified in a subset of IPNBs, predominantly in the intestinal subtype characterized by villous architecture and mucin hypersecretion [105,106,107,108]. *RNF43* mutations are also enriched in the intestinal subtype [106].

#### 3.2.3. Pathological Features

**Figure 5:**
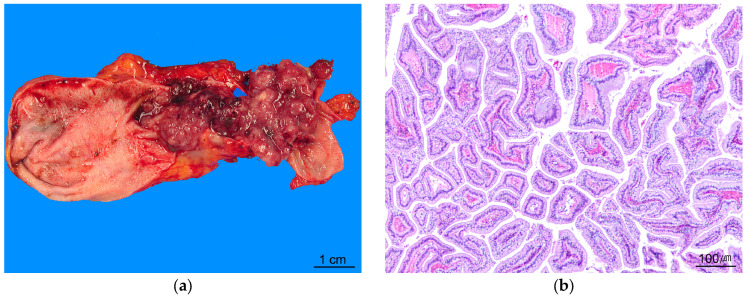
Intraductal papillary neoplasm of the bile duct with low-grade intraepithelial neoplasia. (**a**) A multinodular papillary lesion is present in the extrahepatic biliary tract, including the common hepatic duct, common bile duct, cystic duct, and gallbladder neck. (**b**) The tumor shows a papillary growth pattern lined by tall columnar cells with abundant pale mucin, consistent with gastric foveolar-type differentiation (hematoxylin and eosin stain, ×100).

**Figure 6:**
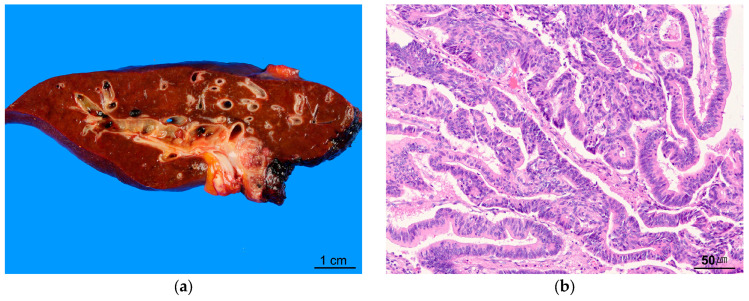
Intraductal papillary neoplasm of the bile duct with high-grade intraepithelial neoplasia. (**a**) A brown solid tumor fills the lumen of the intrahepatic bile duct and is associated with multiple pigmented stones. (**b**) The tumor shows intestinal-type differentiation with a papillary growth pattern characterized by pseudostratified columnar cells with elongated nuclei and marked cytological atypia (hematoxylin and eosin stain, ×200).

**Figure 7:**
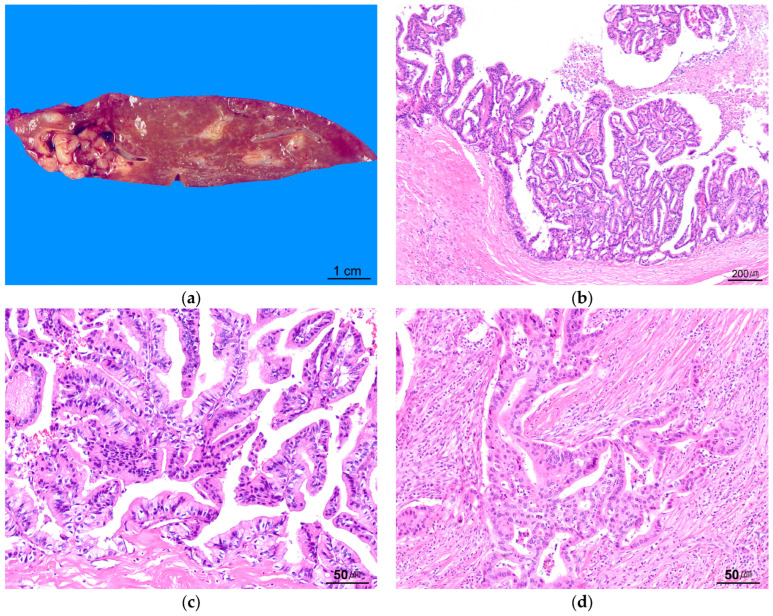
Intraductal papillary neoplasm of the bile duct with associated invasive adenocarcinoma. (**a**) A multinodular lesion is present in the lumen of the left intrahepatic duct. (**b**) The tumor shows a papillary growth pattern lined by biliary-type cuboidal epithelium with low-grade dysplasia (hematoxylin and eosin stain, ×100). (**c**) A transition from low-grade to high-grade dysplasia with marked cytological atypia is present (hematoxylin and eosin stain, ×200). (**d**) An invasive adenocarcinoma component with glandular architecture and desmoplastic stroma is identified (hematoxylin and eosin stain, ×200).

Grossly, IPNBs present as intraluminal polypoid or papillary masses within dilated bile ducts. These dilated ducts may be cystic, cylindrical, or fusiform (mean diameter, 41 ± 22 mm), with papillary projections measuring 5–20 mm [4,105,109]. Lesions may be solitary or multifocal and are often associated with mucin hypersecretion [4,110]. Progression to invasive carcinoma may occur; invasion is usually limited but can occasionally be nodular or mass-forming. Histologically, IPNBs show papillary or villous structures lined by neoplastic epithelium and supported by delicate fibrovascular cores, sometimes with tubular or glandular components [6,105]. IPNBs are graded as low-grade or high-grade dysplasia, with high-grade lesions more frequent in extrahepatic sites [94,105]. IPNBs are subclassified into gastric (Figure 5), intestinal (Figure 6), and pancreaticobiliary (Figure 7) types based on morphology and immunophenotype, with frequent mixed differentiation [105,111,112]. Immunohistochemically, MUC1 expression supports pancreaticobiliary differentiation, MUC2 and CDX2 expression supports intestinal differentiation, and MUC5AC and MUC6 support gastric differentiation [105]. Intestinal and gastric subtypes predominate in Asia, whereas the pancreaticobiliary subtype is more common in Western populations [105]. An associated invasive carcinoma is present in approximately 40–80% of IPNBs at the time of resection, most commonly tubular adenocarcinoma and less frequently colloid carcinoma [6,100,105]. Association with SD-iCCA is rare [105].

IPNB is additionally divided into two types with distinct clinicopathological features [4,99,113]. Type 1 occurs predominantly in intrahepatic ducts, shows cystic or cylindrical dilatation with frequent mucin hypersecretion, and displays a relatively regular papillary architecture with fine fibrovascular cores. It is generally of the gastric or intestinal subtype and is typically high-grade, often with focal low-grade areas. Stromal invasion, when present, is limited. It resembles pancreatic intraductal papillary mucinous neoplasm and generally has a favorable prognosis. Type 2 arises predominantly in extrahepatic ducts, exhibits complex architecture with cribriform or solid areas, and shows minimal mucin production. It is typically of the intestinal or pancreaticobiliary subtype, is almost always high-grade (occasionally with focal low-grade areas), and frequently shows stromal invasion. It behaves more aggressively and is associated with a worse prognosis [92,94,114,115].

#### 3.2.4. Differential Diagnosis

Differential diagnoses include micropapillary BilIN, ITPN, and intraductal polypoid metastases [116,117,118]. Micropapillary BilIN is a microscopic lesion (<3 mm) in the large intrahepatic bile ducts and is often intermixed with flat or pseudopapillary BilIN. ITPN forms a cast-like intraluminal lesion composed of densely packed tubular glands with high-grade architectural and cytological atypia. Mucin production is absent, and MUC5AC expression is typically negative. Intraductal polypoid metastases, most commonly originating from colorectal carcinoma, lack a precursor papillary component and show an abrupt transition to invasive carcinoma.

#### 3.2.5. Key Updates in the 6th WHO Classification

In WHO DST6, IPNB is retained as a grossly visible, noninvasive precursor neoplasm of the large bile ducts. The updated classification formally recognizes the pancreaticobiliary, intestinal, and gastric subtypes, whereas ITPN and IOPN are distinct entities. The type 1/type 2 framework is further clarified: type 2 is characterized by a more complex architecture, invariably high-grade dysplasia, and a stronger association with invasive carcinoma. Molecularly, IPNB pathogenesis follows a stepwise model involving *KRAS* mutations, p16 loss, and *TP53* inactivation, with *GNAS* and *RNF43* mutations being enriched, particularly in the intestinal subtype.

### 3.3. Intraductal Tubulopapillary Neoplasm of the Bile Ducts

#### 3.3.1. Clinical Features

ITPN of the bile ducts is a rare, noninvasive intraductal epithelial neoplasm characterized by tubular structures with focal papillary areas, predominantly high-grade dysplasia, and minimal mucin production. It accounts for approximately 15% of intraductal bile duct neoplasms [93] and shows a female predominance (male-to-female ratio, approximately 1:2), typically occurring in the sixth decade (range, 38–78 years; median, 63 years) [117,119]. Approximately 70% arise in the intrahepatic bile ducts, followed by perihilar (20%) and extrahepatic (10%) locations [117]. Clinically, ITPN may be detected incidentally or present with nonspecific symptoms such as abdominal pain and anorexia, occasionally with weight loss or jaundice [117]. Serum CA19-9 and CEA levels may be elevated. Radiological findings include intraductal filling defects, bile duct dilatation, or a solid mass, occasionally cystic in intrahepatic cases [119,120]. Although the etiology remains unclear, associations with hepatobiliary diseases such as calculi, cirrhosis, PSC, and chronic hepatitis B have been reported [105,121]. Approximately 80% of cases progress to invasive carcinoma, most commonly (approximately 60%) as conventional tubular CCA of small- or large-duct type [105,117]. Despite the high rate of progression to invasive carcinoma, prognosis after resection is excellent, with 1- and 3–5-year survival rates of approximately 100% and 90%, respectively [117]. Even with invasive carcinoma, outcomes remain favorable (5-year survival of approximately 75%), being significantly better than those of conventional CCA [105]. The discrepancy between the high frequency of invasion and the unexpectedly favorable patient outcomes may be attributed to early detection prompted by biliary obstruction or ductal dilatation and to the relatively limited extent of invasive growth in many cases.

#### 3.3.2. Molecular Features

ITPN lacks hotspot mutations in common driver genes of pancreaticobiliary carcinogenesis, such as *KRAS*, *GNAS*, and *IDH1/2*, with only occasional *CDKN2A* alterations reported [93,105,117,122,123]. Instead, low-prevalence alterations in cell cycle regulation, chromatin remodeling (e.g., the SWI/SNF complex, BAP1, and KMT2C), and receptor tyrosine kinase pathways (e.g., FGFR) support a *KRAS*-independent tumorigenic mechanism [93,105,117,123]. Shared chromatin-remodeling gene alterations with CCA suggest partial molecular overlap and a potential biological continuum. Epigenetically, ITPN is further distinguished from IPNB: ITPN has been proposed to arise from MUC6-positive peribiliary glands or cysts [105], whereas IPNB is thought to originate from MUC5AC-positive surface biliary epithelium [120]. MUC5AC-negative biliary ITPNs are genetically distinct from pancreatic ITPNs and IPNBs and may represent a biologically distinct entity from MUC5AC-positive tubular neoplasms, despite their morphological similarities [124].

#### 3.3.3. Pathological Features

**Figure 8:**
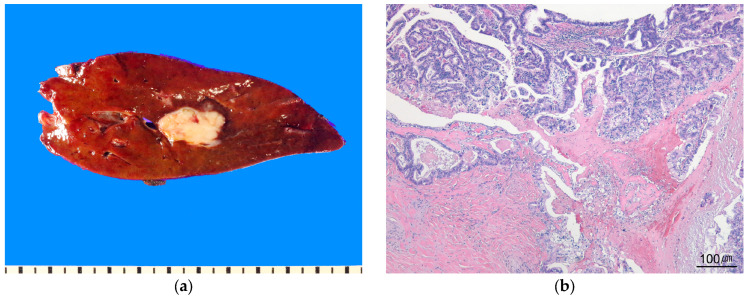
Intraductal tubulopapillary neoplasm of the bile ducts with associated invasive adenocarcinoma. (**a**) A gray-white solid tumor fills the lumen of the intrahepatic bile duct, invades the bile duct wall, and extends into the liver parenchyma. (**b**) The tumor shows a tubulopapillary growth pattern composed of cuboidal cells, with an associated invasive adenocarcinoma component in the lower field (hematoxylin and eosin stain, ×100).

Grossly, ITPNs present as solid, whitish-to-tan nodular masses within dilated bile ducts, often with punctate or geographic necrosis [119,120]. Cyst formation and overt mucin production are uncommon. The mean tumor size is 50 mm (range, 6–150 mm). When invasion occurs, it typically presents as an irregular, firm, whitish mass (Figure 8a). Histologically, biliary ITPNs closely resemble pancreatic ITPNs and show a relatively homogeneous, multinodular architecture composed of tightly packed tubular glands with frequent cribriform formation [93,119,120]. Papillary structures generally comprise <30% of the tumor, and less differentiated solid components may coexist. The tumor cells are cuboidal to columnar, with amphophilic cytoplasm and enlarged nuclei, often with conspicuous nucleoli, consistent with high-grade dysplasia. Nuclear grooves may be present. Mitotic activity is readily identified; additional features may include focal clear or oncocytic changes and occasional intraluminal eosinophilic (colloid-like) secretions, whereas intracytoplasmic mucin is typically absent. Necrosis is common (approximately 85%), usually focal or comedo-like [93,119,120,124]. Invasive carcinoma, when present, most commonly consists of conventional tubular adenocarcinoma of small-duct or large-duct type (Figure 8b). Approximately 25% exhibit an intraductal-like growth pattern with extensive comedonecrosis, and a distinct tubulocystic pattern—characterized by dilated tubular structures lined by bland cells—has been reported [117]. Immunohistochemically, tumor cells are consistently positive for pancytokeratins, CK7, and CK19. MUC1 is expressed in approximately 80% of cases, and MUC6 expression varies (30–100%) [93,117,119], whereas MUC2 and MUC5AC are typically negative [93,117,119].

#### 3.3.4. Differential Diagnosis

Differential diagnoses include BilIN, IPNB, and iCCA. BilIN is distinguished by its flat or micropapillary growth pattern, lack of grossly visible lesions, and predominantly biliary-type morphology. Mucin production, MUC5AC expression, and a heterogeneous spectrum of dysplasia favor IPNB over ITPN [124]. Mass-forming iCCA may extend into large bile ducts and exhibit intraductal tubular growth mimicking ITPN; however, the dominant tumor mass typically resides within the hepatic parenchyma, a feature uncharacteristic of ITPN.

#### 3.3.5. Key Updates in the 6th WHO Classification

Unlike the 5th edition, which described ITPN only as a morphological pattern within IPNB resembling pancreatic ITPN, WHO DST6 formally recognizes biliary ITPN as a distinct entity with well-defined clinicopathological criteria. The updated classification delineates its epidemiology and clinicopathological features, emphasizes its *KRAS*-independent molecular profile characterized by chromatin-remodeling gene alterations, and establishes diagnostic criteria, staging, and prognostic implications.

### 3.4. Intraductal Oncocytic Papillary Neoplasm of the Bile Ducts

#### 3.4.1. Clinical Features

IOPN of the bile ducts is a rare, intraductal epithelial neoplasm characterized by exophytic papillary or nodular proliferation of oncocytic cells with abundant granular eosinophilic cytoplasm. It predominantly affects middle-aged to older adults (range, 38–81 years; mean, 60 years) and shows a slight female predominance [125,126,127,128]. Approximately 70% arise in the intrahepatic bile ducts, whereas the remainder involve the extrahepatic ducts (15%) or both intra- and extrahepatic sites (15%) [127]. IOPN accounts for approximately 9–15% of grossly visible intraductal biliary precursor neoplasms [93,126,127]. Most lesions are incidentally detected, although some patients present with abdominal mass or jaundice [128,129,130]. On imaging, IOPN typically appears as an intraductal papillary or nodular mass within dilated bile ducts and usually lacks diffuse mucin-related ductal dilatation. Associated invasive carcinoma is identified in approximately 30–40% of cases [127,128]. Nevertheless, the overall prognosis is more favorable than that of IPNB-associated invasive carcinoma [127].

#### 3.4.2. Molecular Features

The pathogenesis of biliary IOPN parallels that of pancreatic IOPN and exhibits a distinct molecular profile [131,132,133]. Unlike IPNB, conventional driver mutations (e.g., *KRAS*, *BRAF*, *GNAS*, *RNF43*, *PIK3CA*, and *SMAD4*) are typically absent in IOPN [6,93,134,135]. Instead, IOPNs harbor recurrent gene fusions involving *PRKACA* or *PRKACB*—including *ATP1B1*::*PRKACB*, *ATP1B1*::*PRKACA*, and *DNAJB1*::*PRKACA*—resulting from characteristic chromosomal rearrangements, such as t(1;1)(p31;q24), t(1;19)(q24;p13), and focal chromosome 19 deletion [93,134,136]. These fusions are retained in IOPN-associated invasive carcinomas, supporting their role as early driver alterations [134]. Mechanistically, these fusions activate protein kinase A signaling, leading to mitochondrial hyperplasia and the oncocytic phenotype [137].

#### 3.4.3. Pathological Features

**Figure 9:**
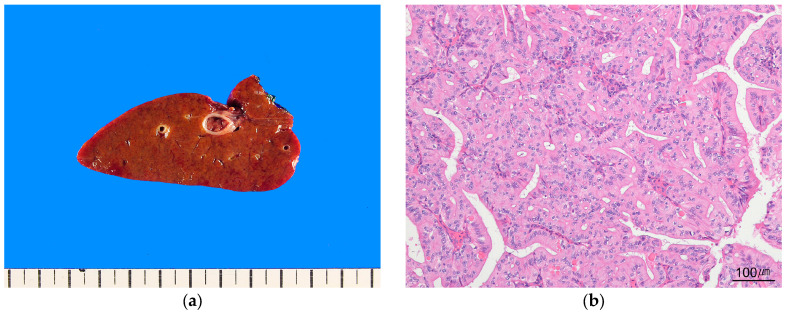
Intraductal oncocytic papillary neoplasm of the bile duct. (**a**) A brown-tan solid tumor fills the lumen of the intrahepatic bile duct. (**b**) The tumor is composed of cuboidal-to-columnar cells with round, uniform nuclei and abundant eosinophilic granular cytoplasm (hematoxylin and eosin stain, ×100).

IOPNs closely resemble pancreatic IOPNs both macroscopically and microscopically [128,133,138,139]. They typically present as large (mean, 53 mm) intraductal masses with friable papillary projections or solid nodules within dilated bile ducts, generally with minimal mucin production (Figure 9a) [112,127,128]. Histologically, biliary IOPNs form complex, arborizing papillae with delicate fibrovascular stroma. The papillae are lined by cuboidal to columnar oncocytic cells with abundant granular eosinophilic cytoplasm, large nuclei, and prominent nucleoli (Figure 9b) [93,140]. They may exhibit cribriform architecture with intraepithelial lumina, scattered goblet cells, and occasional solid growth resulting from papillary fusion. In some cases, oncocytic tumor cells are admixed with gastric foveolar-like tall columnar epithelial cells with subnuclear oncocytic cytoplasm [135,141]. All cases show high-grade dysplasia with architectural complexity and nuclear atypia; inflammatory infiltrates, including lymphocytes and neutrophils, are common. Approximately 30–40% of IOPNs progress to invasive carcinoma, usually composed of small infiltrating tubules or solid nests of oncocytic cells [127,128]. Lymphovascular and perineural invasion, as well as lymph node metastasis, occur at frequencies comparable with those in IPNB-associated carcinoma [127]. Immunohistochemically, tumor cells diffusely express MUC5AC and MUC6 and show focal MUC1 positivity, whereas MUC2 expression is limited to goblet cells [6,93,130,135,142,143]. The mucin expression profiles are similar to those of pancreatic IOPNs [113]. Hepatocyte paraffin 1 (Hep Par-1) and KIT (CD117) are also frequently expressed [127,128].

#### 3.4.4. Differential Diagnosis

Differential diagnoses include IPNB, particularly the pancreaticobiliary subtype, and well-differentiated neuroendocrine tumor (NET). IOPNs are distinguished from IPNBs by their complex cribriform architecture lined by oncocytic cells and diffuse MUC6 expression. Well-differentiated NETs should also be considered, particularly when oncocytic or hepatoid features are present [144,145]. In such cases, neuroendocrine markers (chromogranin A and synaptophysin) should be used alongside IOPN-associated markers (Hep Par-1 and KIT (CD117)) to exclude NET.

#### 3.4.5. Key Updates in the 6th WHO Classification

WHO DST6 formally recognizes biliary IOPN as a distinct entity that shares similar pathogenetic and histopathological features with its pancreatic counterpart. This edition emphasizes its unique molecular profile, characterized by the absence of common driver mutations and the presence of recurrent *PRKACA* and *PRKACB* fusions.

### 3.5. Mass-Forming Intracholecystic Neoplasm

#### 3.5.1. Clinical Features

Mass-forming ICNs are grossly visible, noninvasive polypoid or papillary neoplasms composed of dysplastic epithelium that projects into the gallbladder lumen. According to WHO DST6, mass-forming ICN is a broad category that is histologically subclassified into intracholecystic papillary neoplasm (ICPN) and intracholecystic tubular neoplasm (ICTN) based on their predominant architectural patterns. ICNs account for approximately 0.4% of cholecystectomy specimens; approximately 6% of gallbladder carcinomas arise within them [146]. ICNs predominantly affect adults (mean, 61 years) with a female predominance [146,147,148,149]. Approximately half of patients present with right upper quadrant pain, whereas the remainder are incidentally detected on imaging, often mimicking gallbladder carcinoma [146]. On imaging, ICPN is usually seen as a papillary or polypoid intraluminal mass, whereas ICTN may appear as a compact, sessile, or nodular intraluminal mass. Imaging features such as focal wall thickening, irregular margins, or adjacent hepatic invasion are suggestive of associated invasive carcinoma. ICNs are frequently associated with gallstones [146] and may arise in PSC or PBM [150]. Lesions associated with gallstones or chronic inflammatory conditions are thought to progress through the inflammation–injury–dysplasia–carcinoma sequence, whereas PBM-associated carcinogenesis is preceded by reflux cholecystopathy, defined as mucosal hyperplasia induced by pancreatic enzyme reflux [151]. The prognosis of noninvasive ICNs is excellent after cholecystectomy, whereas those with an invasive component show a 5-year survival of approximately 60% [146].

#### 3.5.2. Molecular Features

ICTNs are primarily characterized by WNT signaling pathway alterations [152,153]. Unlike other pancreaticobiliary intraductal neoplasms, ICNs infrequently harbor canonical driver mutations such as *KRAS* and *GNAS* [148,154,155]; however, *KRAS* mutations occur more frequently in PBM-associated ICNs. *TP53* alterations have been identified in ICPNs [156].

#### 3.5.3. Pathological Features

Grossly, ICNs typically present as granular friable excrescences or exophytic polypoid or papillary mucosal lesions, with a median size of approximately 22 mm [146]. ICPNs are usually broad-based and often extensive, with multifocal lesions identified in approximately one-third of cases, whereas ICTNs are solitary, pedunculated polypoid masses with thin stalks [157]. ICNs arising within adenomyomas form well-demarcated mural nodules that often spare the overlying surface mucosa [158,159].

##### Intracholecystic Papillary Neoplasm

**Figure 10:**
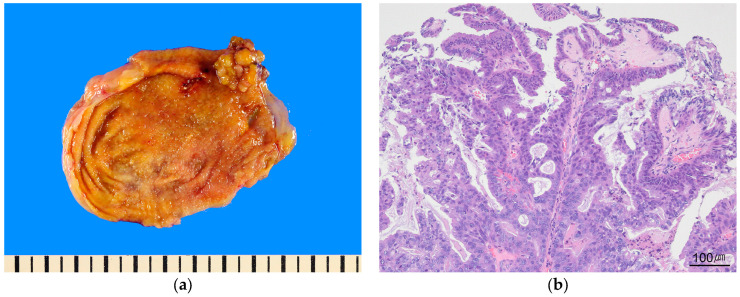
Intracholecystic papillary neoplasm with high-grade intraepithelial neoplasia. (**a**) A polypoid lesion is present in the fundus of the gallbladder. (**b**) The tumor shows a tubulopapillary growth pattern composed of biliary-type cuboidal cells with high-grade dysplasia (hematoxylin and eosin stain, ×100).

ICPNs are broad-based, exophytic lesions composed of neoplastic epithelial cells with predominantly papillary architecture and focal tubular components; they may extend into the Rokitansky–Aschoff sinuses, mimicking invasion. ICPNs show considerable morphologic heterogeneity, most commonly with biliary or gastric differentiation and, less frequently, intestinal differentiation, accompanied by corresponding mucin core protein expression patterns [146,149]. ICPNs are graded as low- or high-grade dysplasia based on the highest degree of cytoarchitectural atypia. High-grade dysplasia is characterized by increased architectural complexity, loss of polarity, and marked nuclear atypia (Figure 10) [146]. Rarely, ICPNs arise within adenomyomas as partly solid and cystic mural nodules; these lesions are usually low-grade, although invasive carcinoma has been reported in approximately 15% of cases [159]. More than half of ICPNs are associated with invasive carcinoma, particularly those with predominantly papillary architecture, biliary differentiation, or extensive high-grade dysplasia [146,149,160]. The invasive component is most often tubular adenocarcinoma; however, mucinous, adenosquamous, and neuroendocrine carcinomas may also occur [161,162].

##### Intracholecystic Tubular Neoplasm

**Figure 11:**
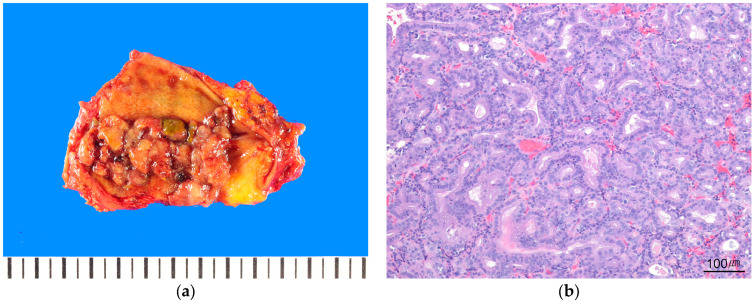
Intracholecystic tubular neoplasm with low-grade intraepithelial neoplasia. (**a**) A large multinodular lesion is present in the body and fundus of the gallbladder. (**b**) Uniform, back-to-back tubules with pyloric gland differentiation are present (hematoxylin and eosin stain, ×100).

ICTNs, previously classified within the intracholecystic papillary–tubular neoplasm spectrum, demonstrate a complex tubular architecture composed of closely packed, back-to-back, acinar-like units lined by mucin-poor epithelial cells with round to ovoid nuclei—a feature that distinguishes them from tubular-predominant ICPNs (Figure 11) [146,150,157,163]. Morule formation and nuclear β-catenin positivity are characteristic. Immunohistochemically, ICTNs are diffusely positive for MUC6 and frequently positive for MUC1, whereas MUC2, MUC5AC, and CDX2 are typically absent [157]. Unlike ICPNs, background mucosal dysplasia is typically absent.

#### 3.5.4. Differential Diagnosis

Differential diagnoses include polypoid pyloric gland metaplasia, IPNB, and invasive carcinoma. ICPNs with gastric differentiation must be distinguished from polypoid pyloric gland metaplasia, which arises in the chronically injured gallbladder as a small polypoid lesion composed of pyloric glands without complex papillary architecture. By definition, ICPN requires either a discrete nodule >10 mm or high-grade dysplasia. IPNB with gastric differentiation arises within the bile ducts and is often associated with mucin hypersecretion and ductal dilatation and frequently harbors *KRAS* and *GNAS* mutations, whereas ICNs exhibit more heterogeneous molecular features. Extension of ICNs into the Rokitansky–Aschoff sinuses may mimic invasion; in contrast, true invasive carcinoma shows irregularly angulated, infiltrative glands with marked cytologic atypia and a desmoplastic stromal reaction.

#### 3.5.5. Key Updates in the 6th WHO Classification

According to WHO DST6, mass-forming intraepithelial neoplasms of the gallbladder are formally designated as ICNs and subclassified into ICPN and ICTN. The term “pyloric gland adenoma (PGA)” is no longer recommended, as these lesions are now largely incorporated into the gastric subtype of ICPNs [2].

Table 3 summarizes the clinicopathological and molecular features of benign biliary tumors and precursor neoplasms in the gallbladder and extrahepatic bile ducts.

## 4. Pathological Diagnostic Approach

Accurate diagnosis of benign biliary tumors and precursor neoplasms requires appropriate specimen handling, adequate sampling, and systematic histopathological evaluation integrated with clinicoradiological findings.

### 4.1. Specimen Handling and Clinicoradiologic Correlation

For biopsy specimens, gross features, including size, color, and consistency, should be carefully documented when available. Specimens should be submitted primarily for routine histopathological examination, with representative tissue retained for ancillary studies as needed. Hematoxylin and eosin (H&E) staining remains the cornerstone of histological evaluation. Serial step sections are recommended to detect subtle architectural abnormalities and to facilitate additional histochemical and immunohistochemical studies if needed. In selected cases, fresh tissue may be submitted for molecular or ultrastructural analysis.

In surgical resection specimens, such as hepatectomy specimens, proper orientation, accurate measurement, and margin assessment are essential. Margins should be identified and inked. Lesions should be evaluated for number, size, color, consistency, presence of necrosis, proximity to margins, and background parenchymal changes. Adequate sampling is critical and should include at least one section per centimeter of tumor, with additional sampling of architecturally heterogeneous regions and tumor–non-neoplastic transition zones. As precursor neoplasms may harbor occult foci of invasion, thorough histological evaluation of all sampled sections is essential to exclude invasive carcinoma [40].

Integration of clinical and radiological information is indispensable for accurate pathological interpretation. Key factors include patient demographics (e.g., female predominance in MCN), lesion location (intrahepatic vs. extrahepatic), imaging features (e.g., ductal communication, cystic architecture, or solid components), and associated conditions (e.g., hepatolithiasis, chronic inflammation). Together, these features provide essential context for narrowing the differential diagnosis.

### 4.2. Histopathological Evaluation

**Figure 12:**
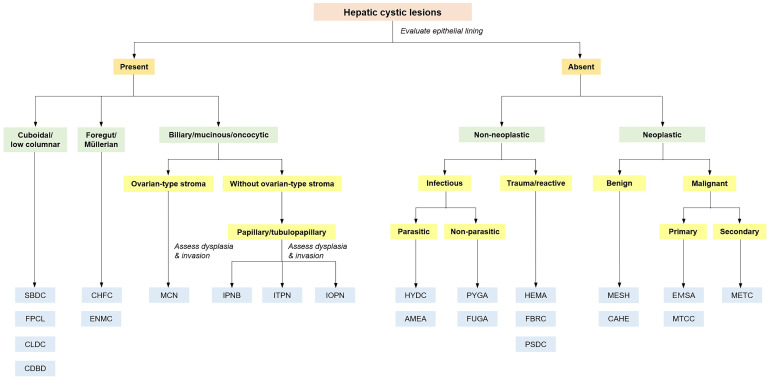
Diagnostic algorithm for hepatic cystic lesions. Hepatic cystic lesions are initially classified according to the presence or absence of an epithelial lining. Epithelial-lined lesions are further categorized by epithelial differentiation into cuboidal/low columnar, foregut/Müllerian, and biliary/mucinous/oncocytic types. Biliary/mucinous/oncocytic lesions are subsequently subdivided according to the presence of ovarian-type stroma and papillary or tubulopapillary proliferation. This facilitates the histopathological differential diagnosis of mucinous cystic neoplasm (MCN), intraductal papillary neoplasm of the bile ducts (IPNB), intraductal tubulopapillary neoplasm (ITPN) of the bile ducts, and intraductal oncocytic papillary neoplasm (IOPN) of the bile ducts. Non-epithelial-lined lesions are classified into non-neoplastic (infectious or traumatic/reactive) and neoplastic (benign or malignant) cystic lesions. AMEA, amebic abscess; CAHE, cavernous hemangioma; CDBD, cystic dilatation of the bile duct; CHFC, ciliated hepatic foregut cyst; CLDC, choledochal cyst; EMSA, embryonal sarcoma of the liver; ENMC, endometriotic cyst; FBRC, fibrous cyst; FPCL, fibropolycystic liver disease; FUGA, fungal abscess; HEMA, hematoma; HYDC, hydatid cyst; MESH, mesenchymal hamartoma; METC, metastatic tumors with cystic change; MTCC, malignant tumors with cystic change; PSDC, pseudocyst; PYGA, pyogenic abscess; SBDC, solitary bile duct cyst. This figure was created by the author solely for illustrative purposes and does not include any patient data or previously unpublished data.

Careful evaluation of H&E-stained sections remains the most critical diagnostic step. At low magnification, the presence of lesional tissue should first be confirmed, followed by evaluation of overall architecture, including circumscription, relationship to adjacent bile ducts, and growth pattern (e.g., nodular, cystic, intraductal, or mass-forming). At high magnification, detailed evaluation of epithelial cytology, degree of atypia, mitotic activity, and stromal features—such as fibrosis, ovarian-type stroma, or inflammatory infiltrates—is essential to distinguish benign lesions from precursor or malignant counterparts. Figure 12 presents a practical histopathological diagnostic algorithm for the differential diagnosis of hepatic cystic lesions based on epithelial lining, morphologic features, and neoplastic potential. When applying this diagnostic algorithm, key histopathological criteria should be evaluated stepwise to facilitate accurate diagnosis. After the initial distinction between epithelial-lined and non-epithelial-lined lesions, epithelial-lined lesions should be further assessed for differentiation type, ductal communication, stromal characteristics, and growth pattern. Ductal communication and intraductal growth favor IPNB, ITPN, or IOPN, whereas ovarian-type stroma in the absence of ductal communication supports the diagnosis of MCN. Architectural pattern, mucin production, oncocytic cytology, and the degree of cytological atypia are essential for further subclassification. Finally, a desmoplastic stromal reaction, irregular invasion, or a dominant parenchymal mass should prompt careful evaluation for associated invasive carcinoma or metastatic disease. The integration of these findings provides a practical framework for the differential diagnosis of hepatic cystic and intraductal biliary lesions. Thus, when the classification of a hepatic cystic lesion is uncertain, the algorithm can be applied in a stepwise manner to refine the differential diagnosis and facilitate lesion categorization.

Despite advances in morphological classification, the evaluation of benign and premalignant biliary lesions remains challenging due to overlapping histologic features and the inability of limited biopsy specimens to adequately represent architectural complexity or stromal invasion. When diagnostic uncertainty persists, a conservative interpretation combined with close clinical follow-up and multidisciplinary correlation should be considered.

**Figure 13:**
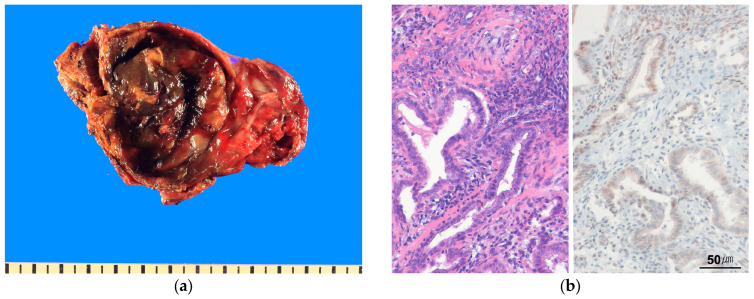
Endometriotic cyst. (**a**) The inner surface of the cystic lesion is brown-tan and hemorrhagic. (**b**) The lesion is composed of epithelium and stroma (**left**). Both epithelial and stromal cells are positive for progesterone receptor (PR) (**right**). PR positivity in glandular epithelial cells is useful for distinguishing an endometriotic cyst from a mucinous cystic neoplasm (hematoxylin and eosin stain [left] and immunohistochemical stain [right], ×200).

**Figure 14:**
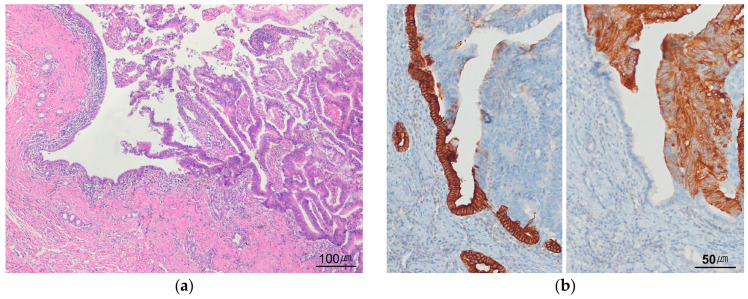
Metastatic colonic adenocarcinoma with intrabiliary growth. (**a**) A transition between the intrabiliary carcinoma and the adjacent non-neoplastic biliary epithelium is present (hematoxylin and eosin stain, ×100). (**b**) The non-neoplastic biliary epithelium is positive for cytokeratin 7 (**left**), whereas the intrabiliary carcinoma is positive for cytokeratin 20 (**right**), supporting the diagnosis of metastatic colonic adenocarcinoma (immunohistochemical stain, ×200).

IHC serves as an important ancillary tool, particularly in diagnostically challenging cases. Markers such as CK7 and CK19 support biliary differentiation, whereas p53, Ki-67, and SMAD4 aid in assessing dysplasia and malignant transformation. PR immunostaining is useful in differentiating endometriotic cysts from MCN (Figure 13). Mucin core proteins (MUC1, MUC2, MUC5AC, and MUC6) and CDX2 help define the epithelial phenotypes of intraductal neoplasms and may support their subclassification into pancreaticobiliary, intestinal, gastric, or pyloric gland differentiation. Furthermore, CK7/CK20 immunohistochemistry aids in distinguishing metastatic colonic adenocarcinoma from primary biliary tumors (Figure 14). In practice, these markers should be selected according to the specific diagnostic question, such as confirming biliary lineage, identifying ovarian-type stroma, subclassifying intraductal neoplasms, evaluating high-grade dysplasia or invasion, or ruling out metastatic disease. However, because no single marker is entirely specific, IHC findings should always be interpreted in the context of histomorphology. Table 4 summarizes the immunohistochemical markers useful for the diagnosis of benign biliary neoplasms and precursor neoplasms.

Molecular analysis provides additional diagnostic and pathogenetic insight in selected cases. Recurrent alterations include *KRAS* and *GNAS* mutations in IPNBs, *PRKACA*/*PRKACB* fusions in IOPNs, and *KRAS*-associated progression in MCNs; in contrast, many benign lesions lack identifiable driver mutations. Accurate diagnosis requires integration of histopathological, immunophenotypic, molecular, and clinicoradiologic findings within a multidisciplinary diagnostic framework.

## 5. Future Perspectives

Several diagnostic challenges highlighted throughout this review remain unresolved, particularly distinguishing REA from BilIN, BDA from well-differentiated SD-iCCA, and BAF from tubulocystic variants of iCCA, especially in limited biopsy specimens. Integration of histopathologic, immunohistochemical, molecular, and radiologic findings will likely become increasingly important for addressing these diagnostic dilemmas and improving the classification and risk stratification of biliary precursor lesions.

Recent advances have substantially improved our understanding of benign biliary tumors and precursor neoplasms; however, their broader molecular landscape and stepwise tumorigenic mechanisms remain incompletely defined. Multi-omics (genomic, epigenomic, and transcriptomic) approaches are expected to refine tumor classification and risk stratification. The tumor immune microenvironment has emerged as a critical component of biliary tumorigenesis; however, its role in biliary precursor neoplasms remains less well defined than in iCCA [164]. A deeper understanding of interactions among the biliary epithelium, stroma, and immune cells may reveal potential targets for chemoprevention and early therapeutic intervention.

Advances in next-generation sequencing (NGS) and spatially resolved omics technologies are expected to further elucidate the molecular evolution from benign lesions to invasive carcinoma. Single-cell and spatial transcriptomic approaches can provide insights into clonal relationships, intralesional heterogeneity, and tumor microenvironmental interactions [165]. From a diagnostic perspective, future biomarker development should focus on identifying highly reproducible ancillary markers that enable reliable distinction between REA and BilIN, as well as between benign biliary lesions and early invasive CCA. For example, integrating NGS-based chromatin-remodeling gene panels and entity-specific fusion analyses may facilitate the classification and risk stratification of biliary precursor neoplasms, particularly as precision oncology continues to expand in hepatobiliary malignancies [166,167]. In parallel, AI-assisted digital pathology platforms may prove particularly valuable in resolving diagnostically challenging cases, especially when evaluating limited biopsy specimens. Such deep-learning approaches have the potential to improve diagnostic reproducibility, efficiency, and workflow management in borderline lesions requiring extensive histologic assessment [168,169,170]. Moreover, recent studies have demonstrated the feasibility of AI-assisted histopathological systems for predicting molecular alterations directly from tumor morphology in iCCA, highlighting their potential role in precision diagnostics [171]. Continued refinement of biliary classification systems will be necessary to harmonize terminology across the pancreaticobiliary system. Close multidisciplinary collaboration among pathologists, clinicians, radiologists, and molecular scientists will be critical to translate these advances into improved patient care and clinical outcomes.

## 6. Conclusions

Benign biliary tumors and precursor neoplasms comprise a heterogeneous group of entities with distinct morphological, immunophenotypic, and molecular features. Their clinical significance lies in their broad biological spectrum, ranging from entirely benign lesions to well-defined precursors of invasive carcinoma. The 2026 WHO classification provides an updated framework for their systematic diagnosis, integrating histopathological, immunophenotypic, and molecular criteria. Further studies are needed to elucidate the molecular mechanisms driving progression to invasive carcinoma and to identify biomarkers for early detection and risk stratification.

## Figures and Tables

**Table 1:** Evolution of the World Health Organization (WHO) classification of benign biliary tumors and precursor neoplasms.

Location	2010 WHO Classification (4th Edition)	2019 WHO Classification (5th Edition)	2026 WHO Classification (6th Edition)
Liver and intrahepatic bile ducts	*Benign*	*Benign biliary tumors and precursors*	*Benign biliary tumors and precursors*
	Bile duct adenoma (peribiliary gland hamartoma and others)	Bile duct adenoma	Bile duct adenoma
	Microcystic adenoma		
	Biliary adenofibroma	Adenofibroma NOS	Biliary adenofibroma
	*Premalignant lesions*		
	BilIN, grade 3	BilIN, low- or high-grade	
	IPNB with low- or intermediate-grade intraepithelial neoplasia	IPNB with low-grade intraepithelial neoplasia	
	IPNB with high-grade intraepithelial neoplasia	IPNB with high-grade intraepithelial neoplasia	
	MCN with low- or intermediate-grade intraepithelial neoplasia	MCN with low-grade intraepithelial neoplasia	MCN with low-grade intraepithelial neoplasia
	MCN with high-grade intraepithelial neoplasia	MCN with high-grade intraepithelial neoplasia	MCN with high-grade intraepithelial neoplasia
	*Malignant*		
	IPNB with an associated invasive carcinoma	IPNB with associated invasive carcinoma	
	MCN with an associated invasive carcinoma	MCN with associated invasive carcinoma	MCN with associated invasive carcinoma
Gallbladder and extrahepatic bile ducts	*Premalignant lesions*	*Benign biliary tumors and precursors*	*Benign epithelial tumors and precursors*
	Adenoma	Adenoma NOS ^(a)^	
	BilIN, grade 3	BBilIN, low- or high-grade	BilIN, low- or high-grade
	Intracystic papillary neoplasm with low- or intermediate-grade intraepithelial neoplasia	Intracystic papillary neoplasm with low-grade intraepithelial neoplasia	
	Intracystic papillary neoplasm with high-grade intraepithelial neoplasia	Intracystic papillary neoplasm with high-grade intraepithelial neoplasia	
	Intraductal papillary neoplasm with low- or intermediate-grade intraepithelial neoplasia	IPNB with low-grade intraepithelial neoplasia	IPNB with low-grade intraepithelial neoplasia
	Intraductal papillary neoplasm with high-grade intraepithelial neoplasia	IPNB with high-grade intraepithelial neoplasia	IPNB with high-grade intraepithelial neoplasia
			ITPN of the bile duct ^(b)^
			IOPN of the bile duct ^(b)^
			Mass-forming intracholecystic neoplasm Intracholecystic papillary neoplasm Intracholecystic tubular neoplasm
	*Carcinoma*		
	Intracystic papillary neoplasm with an associated invasive carcinoma	Intracystic papillary neoplasm with associated invasive carcinoma	
	IPNB with an associated invasive carcinoma	IPNB with associated invasive carcinoma	

NOS, not otherwise specified; BilIN, biliary intraepithelial neoplasia; IPNB, intraductal papillary neoplasm of the bile ducts; MCN, mucinous cystic neoplasm; ITPN, intraductal tubulopapillary neoplasm; IOPN, intraductal oncocytic papillary neoplasm. ^(a)^ Pyloric gland adenoma has been reclassified as the gastric subtype of intracholecystic papillary neoplasm. ^(b)^ ITPN and IOPN are formally recognized as distinct entities in the 6th WHO classification.

**Table 2:** Clinicopathological and molecular features of benign biliary tumors and precursor neoplasms in the liver and intrahepatic bile ducts.

Tumor Types	Clinical Features	Gross Features	Histological Features	Molecular Features	Immunohistochemical Features
Bile duct adenoma	Adults, no sex predilection; approximately 0.6% of autopsy cases; usually presents solitary subcapsular nodule	Small, white, firm, well-circumscribed; typically <10 mm; usually solitary, but can be multiple	Small tubules with cuboidal epithelium, may contain mucin; inflamed or hyalinized fibrous stroma; often include preexisting portal tracts; no infiltrative border	*BRAF* p.V600E mutations in approximately 53% of cases	Positive for CK7, CK19, MUC5AC, MUC6, p16; low Ki-67 index (<10%); CRP and albumin-ISH may be positive
Biliary adenofibroma	Extremely rare; age range, 23–83 years, (median, 57 years), with a slight female predominance; often presents abdominal pain	Well-circumscribed, with solid and microcystic areas; rarely with macrocysts; tumor size can reach up to 160 mm	Tubulocystic structures in fibrotic stroma; cuboidal/low columnar biliary-type cells with apocrine projections; occasional papillary growth; dysplasia or cytological atypia, especially with malignant transformation	*CCND1* and *ERBB2* amplifications associated with aggressiveness; *CDKN2A* (p16) or *NRAS* mutations in rare malignant transformation	Positive for EMA, CK7, CK19, and occasionally CA19-9
Mucinous cystic neoplasm	Adults (mean, 52 years); almost exclusive to women; predominantly intrahepatic; elevated serum and cystic fluid CA19-9 and CEA levels	Multilocular cystic lesion (mean 110 mm); smooth or trabeculated inner wall, with papillary projections; mucinous, clear, or hemorrhagic fluid	Cysts lined by columnar, cuboidal, or flattened epithelium with mucinous or pale eosinophilic cytoplasm and basally oriented nuclei; ovarian-type stroma; inflammation and degenerative changes	*KRAS* mutation (approximately 20%); telomere shortening with progression to invasive carcinoma	Positive for CK7, CK8, CK18, CK19, EMA, CEA, and MUC5AC (epithelial cells); positive for ER, PR, and α-inhibin (ovarian-type stromal cells)

CK, cytokeratin; MUC, mucin core protein; CRP, C-reactive protein; ISH, in situ hybridization; EMA, epithelial membrane antigen; CA19-9, carbohydrate antigen 19-9; CEA, carcinoembryonic antigen; ER, estrogen receptor; PR, progesterone receptor.

**Table 3:** Clinicopathological and molecular features of benign biliary tumors and precursor neoplasms in the gallbladder and extrahepatic bile ducts.

Tumor Types	Clinical Features	Gross Features	Histological Features	Molecular Features	Immunohistochemical Features
BilIN	Associated with hepatolithiasis, cholelithiasis, PSC, choledochal cysts, AUPBD, and parasitic infections	Usually not visible; subtle mucosal thickening or textural change	Low-grade: flat, mild atypia, hyperchromasia, preserved polarity, rare mitoses; high-grade: complex (micropapillary or tall papillary) architecture, loss of polarity, marked atypia, frequent mitoses	DNA repair gene alterations (*ATM*, *BRCA2*); tumor suppressor gene hypermethylation; mutations in *CTNNB1*, *TP53*, *KRAS*; *CTNNB1* alterations associated with invasion	Positive for CK7, CK19; p53 overexpression (high-grade)
Intraductal papillary neoplasm of the bile ducts	Middle-aged to elderly patients (42–80 years); slight male predominance; abdominal pain, jaundice, recurrent cholangitis; risk factors ^(a)^	Intraluminal polypoid or papillary masses within dilated bile ducts; solitary or multifocal; often mucin hypersecretion	Papillary or villous structures with delicate fibrovascular cores; graded as low- or high-grade; intestinal, pancreaticobiliary, or gastric types, often with mixed differentiation	*KRAS* mutations; *TP53* inactivating mutations; *GNAS* mutations (intestinal type with villous pattern and mucin hypersecretion); *RNF43* mutations (intestinal type)	Positive for MUC1 (pancreaticobiliary type), MUC2, CDX2 (intestinal type), MUC5AC (gastric foveolar type), MUC6 (pyloric gland type)
Intraductal tubulopapillary neoplasm of the bile ducts	Age (38–78 years; median, 63 years); female predominance; incidental or present with abdominal pain, anorexia, weight loss, or jaundice	Whitish-to-tan nodular masses filling dilated bile ducts; mean size ~50 mm (6–150 mm); firm, irregular masses in invasive lesions	Multinodular, tightly packed tubular glands with cribriform architecture; predominantly high-grade dysplasia with amphophilic cytoplasm, nuclear atypia, mitoses; mucin production absent; necrosis common	*CDKN2A* (p16) alterations; chromatin-remodeling gene alterations (SWI/SNF complex genes, *BAP1*, *KMT2C*); receptor tyrosine kinase pathway alterations	Positive for pan-CK, CK7, CK19; MUC1 (80%); MUC6 (30–100%)
Intraductal oncocytic papillary neoplasm of the bile ducts	Middle-aged to older adults (38–81 years, mean, 60 years); slight female predominance; often incidental, occasionally abdominal mass or jaundice	Large (~53 mm), tan-brown intraductal mass with friable papillary projections or solid nodules; minimal mucin production	Complex arborizing papillae lined by oncocytic cells with eosinophilic granular cytoplasm, large nuclei, and prominent nucleoli; cribriform structures; uniform cells; high- grade dysplasia	Recurrent gene fusions involving *PRKACA* or *PRKACB* (e.g., *ATP1B1*::*PRKACB*, *ATP1B1*::*PRKACA*, *DNAJB1*::*PRKACA*)	Positive for MUC5AC and MUC6 (diffuse); MUC1 (focal); MUC2 limited to goblet cells; Hep Par-1 and KIT (CD117) (frequently)
Mass-forming intracholecystic neoplasm	Wide age range (mean 61 years); female predominance; often associated with gallstones, PSC, or PBM; incidentally or often right upper quadrant pain	ICPN: exophytic or polypoid lesions; broad-based, often multifocal (~1/3 cases). ICTN: solitary, pedunculated polyps with thin stalks.	ICPN: papillary architecture with focal tubular components; low- to high-grade; biliary, gastric, or intestinal differentiation. ICTN: complex tubular structures of mucin-poor cells and morules.	ICPN: *TP53* alterations, uncommon *KRAS* mutations (except in PBM-associated cases), rare *GNAS* mutations. ICTN: *CTNNB1* alterations, WNT pathway activation	ICPN: Subtype-dependent MUC expression. ICPN: MUC1 and MUC6 expression

BilIN, biliary intraepithelial neoplasia; PSC, primary sclerosing cholangitis; AUPBD, anomalous union of the pancreaticobiliary ducts; CK, cytokeratin; MUC, mucin core protein; CDX2, caudal type homeobox 2; Hep Par-1, hepatocyte paraffin 1; KIT, KIT proto-oncogene receptor tyrosine kinase; PBM, pancreaticobiliary maljunction; ICPN, intracholecystic papillary neoplasm; ICTN, intracholecystic tubular neoplasm. ^(a)^ Risk factors include primary sclerosing cholangitis, hepatolithiasis, liver fluke infection (*Clonorchis sinensis, Opisthorchis viverrini*), and pancreaticobiliary maljunction.

**Table 4:** Immunohistochemical markers for the diagnosis of benign biliary tumors and precursor neoplasms of the bile ducts.

Immunohistochemical Markers	Approximate Frequency	Comment
Biliary differentiation markers		
CK (AE1/AE3)	~100%	Pan-epithelial marker; widely expressed in biliary epithelial neoplasms
CK7	95–100%	Sensitive and consistently expressed marker of biliary epithelium; positive in BDA, BAF, MCN, BilIN, IPNB, ITPN, and IOPN
CK19	90–100%	Supports biliary ductal differentiation; co-expressed with CK7 in most biliary neoplasms
EMA	70–90%	Apical/luminal membrane staining; highlights glandular differentiation; strong expression in pancreaticobiliary-type IPNB and ITPN; often weak or focal in BDA
MUC phenotype markers		
MUC1	Variable ^(a)^	Pancreaticobiliary-type differentiation; associated with invasive/aggressive phenotype; frequently expressed in pancreaticobiliary-type IPNB (~80%); variably expressed in ITPN; absent or weak in BDA
MUC2	Variable ^(a)^	Intestinal differentiation (goblet cell lineage); positive in intestinal-type IPNB (70–100%); expression typically limited to goblet cells in IOPN; negative in ITPN; usually absent in BDA and BAF
MUC5AC	Variable ^(a)^	Gastric foveolar differentiation; positive in gastric-type IPNB (~80–90%); negative in ITPN; may be expressed in IOPN; may be useful for distinguishing IPNB from ITPN
MUC6	Variable ^(a)^	Pyloric gland differentiation; positive in gastric-type IPNB (30–80%); diffusely positive in IOPN; variably expressed in BDA and ITPN
Intestinal differentiation markers		
CDX2	Variable ^(a)^	Nuclear marker of intestinal differentiation; frequently positive in intestinal-type IPNB (50–80%); expression may be limited to goblet cells in IOPN; negative in ITPN, BDA, and BAF
SATB2	Variable ^(a)^	May support intestinal differentiation; but is not routinely used for biliary precursor neoplasms; primarily useful in excluding colorectal origin; positive in intestinal-type IPNB; negative in ITPN
Hormone receptor marker		
ER	70–100%	Positive in ovarian-type stromal cells of MCN; supports the diagnosis of MCN; negative in BDA, BAF, IPNB, and ITPN
PR	80–100%	Positive in ovarian-type stromal cells of MCN; supports MCN diagnosis alongside ER; often more consistently expressed than ER in MCN stroma
CD10	50–80%	Positive in stromal cells of MCN; may be positive in epithelial cells in MCN
α-inhibin	30–70%	Positive in luteinized stromal cells of MCN; helpful in confirming ovarian-type stroma
Markers for distinguishing benign and malignant biliary tumors		
p53	Variable abnormal expression	Overexpression (>50% strong nuclear staining) or null/aberrant pattern supports malignancy or high-grade dysplasia; wild-type (scattered weak positive) pattern in BDA, BAF (low-grade), and VMC; may be useful in distinguishing BilIN from reactive atypia
SMAD4	Loss in 30–50% (carcinoma)	Loss of nuclear expression supports carcinoma (especially in iCCA); retained in BDA, BAF, and noninvasive biliary precursors
S100P	60–90% (carcinoma)	Expressed in carcinoma; negative in BDA and BAF; may assist in distinguishing benign from malignant biliary lesions
EZH2	50–80% (carcinoma)	Expressed in high-grade dysplasia and carcinoma; typically absent in benign lesions, including BDA and BAF; useful marker for distinguishing BDA from iCCA
p16	Loss in 30–70% (carcinoma)	Retained expression in BDA and benign biliary lesions; loss of expression supports carcinoma; homozygous deletion of *CDKN2A* has been identified in BAF with malignant transformation
Ki-67 labeling index	<10% (benign); >20–30% (carcinoma)	Low proliferative index in BDA (<10%) and BAF epithelium (<10%); elevated in high-grade dysplasia and invasive carcinoma; useful adjunct to p53 and EZH2 in distinguishing BDA from iCCA
Others		
Hep Par-1	20–50% (IOPN)	Expressed in IOPN; usually negative in most other biliary precursor neoplasms; positivity may mimic hepatocellular differentiation
KIT (CD117)	20–60% (IOPN)	Expressed in IOPN; useful supportive marker when combined with oncocytic morphology, MUC5AC/MUC6, and Hep Par-1; usually negative in IPNB, ITPN, BDA, and BAF
CA19-9	50–80% (BAF, MCN)	Expressed in BAF and MCN, supporting biliary phenotype; serum and cyst fluid CA19-9 levels may be elevated in MCN but are not specific
CEA	60–90% (MCN)	Expressed in MCN epithelium; elevated cystic fluid CEA may support MCN but is not diagnostic alone
CRP	30–70% (BDA)	Expressed in BDA, but also expressed in iCCA; interpretation should be integrated with morphology and other markers
Albumin ISH	50–80% (BDA)	Expressed in BDA; usually negative or markedly reduced in iCCA

CK, cytokeratin; BDA, bile duct adenoma; BilIN, biliary intraepithelial neoplasia; BAF, biliary adenofibroma; MCN, mucinous cystic neoplasm; IPNB, intraductal papillary neoplasm of the bile duct; ITPN, intraductal tubulopapillary neoplasm; IOPN, intraductal oncocytic papillary neoplasm; MUC, mucin core protein; EMA, epithelial membrane antigen; CDX2, caudal type homeobox 2; SATB2, SATB2 homeobox 2; ER, estrogen receptor; PR, progesterone receptor; VMC, von Meyenburg complex; SMAD4, SMAD family member 4; S100P, S100 calcium binding protein P; EZH2, enhancer of zeste homolog 2; iCCA, intrahepatic cholangiocarcinoma; Hep Par-1, hepatocyte paraffin 1; KIT, KIT proto-oncogene receptor tyrosine kinase; CA19-9, carbohydrate antigen 19-9; CEA, carcinoembryonic antigen; CRP, C-reactive protein; ISH, in situ hybridization. ^(a)^ Expression frequency varies among studies, histologic subtypes, and diagnostic criteria; exact percentages should therefore be interpreted with caution.

## Data Availability

All data were included in the manuscript.

## Abbreviations

The following abbreviations are used in this manuscript:

AI	Artificial intelligence
AMEA	Amebic abscess
*APC*	Adenomatous polyposis coli
*ARID1A*	AT-rich interaction domain-containing protein 1A
*ARID2*	AT-rich interaction domain-containing protein 2
*ATM*	Ataxia telangiectasia mutated
AUPBD	Anomalous union of the pancreaticobiliary ducts
BAF	Biliary adenofibroma
BAP1	BRCA1-associated protein 1
BDA	Bile duct adenoma
BilIN	Biliary intraepithelial neoplasia
*BRCA2*	Breast cancer susceptibility gene 2
CA19-9	Carbohydrate antigen 19-9
CAHE	Cavernous hemangioma
CCA	Cholangiocarcinoma
CD117	Cluster of differentiation 117
CDBD	Cystic dilatation of the bile duct
*CDH1*	Cadherin 1
*CDKN2A*	Cyclin-dependent kinase inhibitor 2A
CDX2	Caudal type homeobox 2
CEA	Carcinoembryonic antigen
CHFC	Ciliated hepatic foregut cyst
CK	Cytokeratin
CLDC	Choledochal cyst
CRP	C-reactive protein
*CTNNB1*	Catenin beta 1
*DCC*	Deleted in colorectal carcinoma
*DNAJB1*	DnaJ heat shock protein family (Hsp40) member B1
DR	Ductular reaction
EMA	Epithelial membrane antigen
EMSA	Embryonal sarcoma of the liver
ENMC	Endometriotic cyst
ER	Estrogen receptor
*ERBB2*	Erb-b2 receptor tyrosine kinase 2
*ERBB3*	Erb-b3 receptor tyrosine kinase 3
EZH2	Enhancer of zeste homolog 2
FBRC	Fibrous cyst
FGFR	Fibroblast growth factor receptor
*FGFR2*	Fibroblast growth factor receptor 2
*FHIT*	Fragile histidine triad
FPCL	Fibropolycystic liver disease
FUGA	Fungal abscess
*GNAS*	Guanine nucleotide-binding protein, alpha-stimulating
H&E	Hematoxylin and eosin
HEMA	Hematoma
Hep Par-1	Hepatocyte paraffin 1
HYDC	Hydatid cyst
iCCA	Intrahepatic cholangiocarcinoma
ICN	Intracholecystic neoplasm
ICPN	Intracholecystic papillary neoplasm
ICTN	Intracholecystic tubular neoplasm
*IDH*	Isocitrate dehydrogenase
*IDH1*	Isocitrate dehydrogenase 1
*IDH2*	Isocitrate dehydrogenase 2
IHC	Immunohistochemistry
IOPN	Intraductal oncocytic papillary neoplasm
IPNB	Intraductal papillary neoplasm of the bile ducts
ISH	In situ hybridization
ITPN	Intraductal tubulopapillary neoplasm
KIT	KIT proto-oncogene receptor tyrosine kinase
Ki-67	Ki-67 antigen
KMT2C	Lysine methyltransferase 2C
*KRAS*	*KRAS* proto-oncogene, GTPase
LD-iCCA	Large-duct type intrahepatic cholangiocarcinoma
MCN	Mucinous cystic neoplasm
MESH	Mesenchymal hamartoma
METC	Metastatic tumors with cystic change
MTCC	Malignant tumors with cystic change
MUC	Mucin
MUC1	Mucin 1
MUC2	Mucin 2
MUC5AC	Mucin 5AC
MUC6	Mucin 6
N:C ratio	Nuclear-to-cytoplasmic
NET	Neuroendocrine tumor
*NFE2L2*	Nuclear factor erythroid 2-related factor 2
NGS	Next-generation sequencing
*NRAS*	*NRAS* proto-oncogene, GTPase
PBM	Pancreaticobiliary maljunction
*PBRM1*	Polybromo 1
PGA	Pyloric gland adenoma
PIK3CA	Phosphatidylinositol-4,5-bisphosphate 3-kinase catalytic subunit alpha
PR	Progesterone receptor
*PRKACA*	Protein kinase cAMP-activated catalytic subunit alpha
*PRKACB*	Protein kinase cAMP-activated catalytic subunit beta
PSC	Primary sclerosing cholangitis
PSDC	Pseudocyst
PYGA	Pyogenic abscess
RB1	RB transcriptional corepressor 1
REA	Reactive epithelial atypia
*RNF43*	Ring finger protein 43
S100P	S100 calcium-binding protein P
SATB2	SATB homeobox 2
SBDC	Solitary bile duct cyst
SD-iCCA	Small-duct type intrahepatic cholangiocarcinoma
SMAD4	SMAD family member 4
*SMARCA4*	SWI/SNF-related, matrix-associated, actin-dependent regulator of chromatin subfamily A member 4
SWI/SNF	SWItch/Sucrose Non-Fermentable chromatin-remodeling complex
*TP53*	Tumor protein p53
VMC	Von Meyenburg complex
WHO	World Health Organization
WHO DST6	Sixth edition World Health Organization Classification of Digestive System Tumours
